# Real-Time Electronic Patient Portal Use Among Emergency Department Patients

**DOI:** 10.1001/jamanetworkopen.2024.9831

**Published:** 2024-05-03

**Authors:** Robert W. Turer, Samuel A. McDonald, Christoph U. Lehmann, Bhaskar Thakur, Sayon Dutta, Richard A. Taylor, Christian C. Rose, Adam Frisch, Kristian Feterik, Craig Norquist, Carrie K. Baker, Jeffrey A. Nielson, David Cha, Brian Kwan, Christian Dameff, James P. Killeen, Michael K. Hall, Robert C. Doerning, S. Trent Rosenbloom, Casey Distaso, Bryan D. Steitz

**Affiliations:** 1 Department of Emergency Medicine, University of Texas Southwestern Medical Center, Dallas; 2 Clinical Informatics Center, University of Texas Southwestern Medical Center, Dallas; 3 Department of Pediatrics, University of Texas Southwestern Medical Center, Dallas; 4 Department of Emergency Medicine, Mass General Brigham, Boston, Massachusetts; 5 Mass General Brigham Digital, Boston, Massachusetts; 6 Department of Emergency Medicine and Section for Biomedical Informatics and Data Science, Yale School of Medicine, New Haven, Connecticut; 7 Department of Biostatistics, Yale School of Public Heath, New Haven, Connecticut; 8 Department of Emergency Medicine, Stanford University School of Medicine, Stanford, California; 9 Department of Emergency Medicine, University of Pittsburgh School of Medicine, Pittsburgh, Pennsylvania; 10 Department of Emergency Medicine, HonorHealth, Phoenix, Arizona; 11 Department of Emergency Medicine, Kettering Health, and Wright State University Boonshoft School of Medicine, Dayton, Ohio; 12 Department of Emergency Medicine, School of Medicine, University of California, San Diego; 13 Department of Emergency Medicine, University of Washington, Seattle; 14 Departments of Internal Medicine and Pediatrics, Vanderbilt University Medical Center, Nashville, Tennessee; 15 Department of Biomedical Informatics, Vanderbilt University Medical Center, Nashville, Tennessee

## Abstract

**Importance:**

Patients with inequitable access to patient portals frequently present to emergency departments (EDs) for care. Little is known about portal use patterns among ED patients.

**Objectives:**

To describe real-time patient portal usage trends among ED patients and compare demographic and clinical characteristics between portal users and nonusers.

**Design, Setting, and Participants:**

In this cross-sectional study of 12 teaching and 24 academic-affiliated EDs from 8 health systems in California, Connecticut, Massachusetts, Ohio, Tennessee, Texas, and Washington, patient portal access and usage data were evaluated for all ED patients 18 years or older between April 5, 2021, and April 4, 2022.

**Exposure:**

Use of the patient portal during ED visit.

**Main Outcomes and Measures:**

The primary outcomes were the weekly proportions of ED patients who logged into the portal, viewed test results, and viewed clinical notes in real time. Pooled random-effects models were used to evaluate temporal trends and demographic and clinical characteristics associated with real-time portal use.

**Results:**

The study included 1 280 924 unique patient encounters (53.5% female; 0.6% American Indian or Alaska Native, 3.7% Asian, 18.0% Black, 10.7% Hispanic, 0.4% Native Hawaiian or Pacific Islander, 66.5% White, 10.0% other race, and 4.0% with missing race or ethnicity; 91.2% English-speaking patients; mean [SD] age, 51.9 [19.2] years). During the study, 17.4% of patients logged into the portal while in the ED, whereas 14.1% viewed test results and 2.5% viewed clinical notes. The odds of accessing the portal (odds ratio [OR], 1.36; 95% CI, 1.19-1.56), viewing test results (OR, 1.63; 95% CI, 1.30-2.04), and viewing clinical notes (OR, 1.60; 95% CI, 1.19-2.15) were higher at the end of the study vs the beginning. Patients with active portal accounts at ED arrival had a higher odds of logging into the portal (OR, 17.73; 95% CI, 9.37-33.56), viewing test results (OR, 18.50; 95% CI, 9.62-35.57), and viewing clinical notes (OR, 18.40; 95% CI, 10.31-32.86). Patients who were male, Black, or without commercial insurance had lower odds of logging into the portal, viewing results, and viewing clinical notes.

**Conclusions and Relevance:**

These findings suggest that real-time patient portal use during ED encounters has increased over time, but disparities exist in portal access that mirror trends in portal usage more generally. Given emergency medicine’s role in caring for medically underserved patients, there are opportunities for EDs to enroll and train patients in using patient portals to promote engagement during and after their visits.

## Key Points

**Question:**

During emergency department (ED) visits, what proportion of patients actively accesses and views their health information in real time through the patient portal?

**Findings:**

In this cross-sectional study of 1 280 924 adult patients presenting at 36 EDs across the US between April 5, 2021, and April 4, 2022, 17.4% logged into the patient portal while in the ED. Although patients increasingly used the portal, lower odds of accessing the portal were observed among patients who were male, Black, or without commercial insurance.

**Meaning:**

These findings suggest opportunities for EDs to promote patient engagement in portal use during ED encounters.

## Teaser

This cross-sectional study evaluates patients’ portal usage trends while in the emergency department and compares demographic and clinical characteristics between users and nonusers.

## Introduction

Patients and care partners increasingly use patient portals to understand and manage their health and health care. Among a 2022 national sample of adults, 77% had been offered access to a patient portal and 68% accessed one, an increase from 37% and 31%, respectively, in 2014.^1^ The 21st Century Cures Act’s information blocking provision,^2^ which spurred the widespread practice of information sharing since 2021, may have contributed to this increase. Since this provision, most health records, including clinical notes, test results, and medical history, have become available to patients and care partners via portals upon publication to the electronic health record (EHR).^2,3,4,5^ Despite increased adoption, disparities persist in portal use among patients who are older, are Black or Hispanic, have low health or digital literacy, or prefer non-English languages.^6^

The information blocking provision applies uniformly across care settings, including acute care settings such as emergency departments (EDs). Information sharing in these task-saturated environments may create unique challenges for patient engagement, expectation management, and precounseling strategies for high-stakes tests such as HIV screening or cancer reconnaissance. Literature about ED portal use is sparse,^7,8,9,10^ which is problematic because the ED represents the primary care environment for many medically underserved and underinsured patients who use portals at lower rates despite equally or more complex care needs.^6,11^ Preliminary studies suggested that portal access initiated in the ED, especially among patients experiencing houselessness, is associated with increased clinic attendance and care adherence.^12,13,14^ Improved care adherence may follow from the ability to manage care asynchronously by scheduling appointments, messaging care teams, and reviewing results, which extends the care network beyond the doors of the ED.^15,16^ More acutely, real-time ED portal access might facilitate sooner identification of critical results or empower patients to detect missed incidental findings.^17^

The literature on ED patients reviewing results is meager. Foster and Krasowski^15^ found that nearly 9% of patients who arrived at the ED between 2016 and 2017 with active portal accounts reviewed results within 72 hours of their visit. There remains a need for robust, representative knowledge about real-time portal usage in EDs across the US. To fulfill this need and provide more generalizable results, we conducted a multisite study to measure trends in real-time portal usage among ED patients since the Cures Act information blocking provision and to compare clinical and demographic characteristics between portal users and nonusers.

## Methods

### Study Design, Setting, and Population

We conducted a cross-sectional study to evaluate trends and characteristics of real-time portal use among patients seen in 12 teaching and 24 academic-affiliated community EDs representing 8 health systems in California, Connecticut, Massachusetts, Ohio, Tennessee, Texas, and Washington. Sites were recruited on a voluntary basis from the Society for Academic Emergency Medicine’s Informatics, Data Science, and Artificial Intelligence interest group based on interest, available technical expertise, and data availability (ie, test results were available for viewing during ED visits). To facilitate parallel data extraction, we only recruited sites that used the Epic EHR and MyChart patient portal (Epic Systems Corporation). eTable 1 in Supplement 1 lists the site characteristics. The institutional review board at each site approved the study procedures and granted waivers of informed consent. Waivers of consent were granted due to the impracticality of obtaining consent from a cohort of over a million patients, especially given the use of retrospective EHR data collected during routine care. We followed the Reporting of Studies Conducted Using Observational Routinely-Collected Health Data (RECORD) reporting guidelines.^18^

We included all patients aged 18 years or older presenting to participating EDs between April 5, 2021, and April 4, 2022, representing the first year since the Cures Act information blocking provision took effect. At all participating sites, patients could access the portal in real time if they had a MyChart account prior to arrival or if they enrolled during the visit. Enrollment tokens could be sent to patients by registration staff, clinicians, or nurses at any time during an ED encounter via text, email, or printed after-visit summary, depending on how each system was configured.

All sites extracted portal use, demographic, and clinical data using Epic’s Clarity reporting database. There were no external data linkages.

### Framework for Multisite Analysis

We conducted this study using semifederated data extraction and analysis. This approach facilitated rapid recruitment of sites from across the country, avoided the need for cross-site institutional review board and data use agreements, and allowed sites to participate without sharing patient-level data. Each site extracted its own data and computed descriptive statistics and multivariable models. All sites extracted data using the same Epic Clarity query with identical variable structure, which ensured data consistency and facilitated downstream aggregation. Some query fields required local mapping at each site (eg, fields describing race, ethnicity, language, procedure types, and note types). Guides describing the mapping process from the coordinating site (University of Texas Southwestern Medical Center) facilitated data consistency. Board-certified physician informaticians (R.W.T., S.A.M., C.U.L., S.D., R.A.T., C.C.R., K.F., C.N., C.K.B., J.A.N., C. Dameff, J.P.K., R.C.D., and C. Distaso) at each site with expertise in the ED and MyChart Clarity data models reviewed local data and attended biweekly meetings to address inconsistencies. Sites used identical R, version 4.3.2 (R Project for Statistical Computing) scripts to provide consistent results structures.^19^ Each site shared descriptive statistics and modeling results with the coordinating site to aggregate descriptive statistics and fit pooled models.

### Measures

The outcome variable of interest was the weekly proportion of ED patients who used the portal as defined by logging into the system, viewing test results, or viewing clinical notes between ED arrival and departure (ie, discharge, transfer, hospital admission). We collected demographic variables, including age at arrival, sex, insurance status during the encounter, preferred language, ethnicity, and race. Clinical variables included Emergency Severity Index (ESI) and the patient’s MyChart account activation status within the health system prior to ED arrival.

We grouped age into the following categories: 18 to 34 years, 35 to 49 years, 50 to 64 years, 65 to 84 years, and 85 years or older. Gender data, including nonbinary gender identification, were not consistently available among sites, so we used Epic’s legal sex field. We classified primary insurance status as commercial (including commercial, agency, exchange, and managed care), Medicaid, Medicare, self-pay (including Medicaid pending), or other (including workers’ compensation or other nonclassified payers). We report the following primary language categories: English, Spanish, or other. Because of substantial variability among sites regarding which languages were selectable within Epic, English and Spanish were the only languages at sufficient rates at all sites to facilitate downstream analysis using identical models.

According to the National Academy of Medicine’s proposed approach,^20,21^ we included the following concepts as dichotomous, potentially co-occurring variables representing race and ethnicity as documented in the EHR: American Indian or Alaska Native, Asian, Black or African American, Hispanic or Latino, Native Hawaiian or Pacific Islander, White, and other. Epic’s ED registration workflow and question structure follows the US Office of Management and Budget 2-question approach for capturing ethnicity (ethnic group variable in Epic) and race by default.^20^ Several sites expanded their EHR’s ethnicity or race data structures to include other classifications; we consolidated these to the previously described categories to allow for uniform reporting. Patients can simultaneously report multiple ethnicity and race classifications in the EHR, which is reflected in our data sets and analyses. Racial and ethnic disparities have been associated with lower rates of portal use in prior studies.^1^ Given the diverse population of patients who visit EDs, it is critical to understand whether those disparities exist within the emergency care environment as well.

The ESI is a widely recognized acuity score that ranges from 1 (highest acuity) to 5 (lowest acuity) and is typically performed by ED nurses during triage.^22^ Emergency department dispositions were classified as admission (including both inpatient and observation stays), discharge, or other (including patients who left without being seen, before completion of care, or against medical advice or patients who immediately went to labor and delivery).

### Statistical Analysis

We report descriptive statistics comparing characteristics of patients who used and did not use the portal during their ED visit, stratified by activity: logging into MyChart, viewing test results, and viewing clinical notes. To assess temporal trends, we plotted weekly proportions of patients engaging in each activity by site and overall.

Each site fit 3 exploratory multivariable binary logistic regression models, 1 for each activity, to identify associations between patient characteristics and the likelihood of each activity. We included the following variables in each model: time (week of study), age, ESI, sex, race, ethnicity, preferred language, insurance status, MyChart activation status at arrival, and disposition. To account for patients with multiple encounters, each site performed cluster-robust SE adjustment using the vcovCL function in the sandwich R package.^23^ Each site performed random forest imputation using the missRanger R package.^24^

We used effect size estimates and SEs from each site’s multivariable model to create random-effects models using the metafor meta-analysis R package.^25^ We report pooled odds ratios (ORs) with 95% CIs as well as prediction intervals for each variable. Prediction intervals describe the interval within which most true effects are expected to fall and are considered better practice for assessing intersite heterogeneity than *Q* tests or *I*^2^ statistics.^26,27^ We calculated prediction intervals using metafor’s predict.rma function. An α of .05 was set for significance testing.

## Results

We observed 1 280 924 ED encounters (mean [SD] age, 51.9 [19.2] years; 53.5% women and 46.5% men). Encounters most frequently involved patients who were White (66.5%, compared with 0.6% American Indian or Alaska Native, 3.7% Asian, 18.0% Black, 10.7% Hispanic, 0.4% Native Hawaiian or Pacific Islander, 10.0% other race, and 4.0% with missing data), English speaking (91.2% compared with 5.4% Spanish speaking and 2.9% other language preference), and commercially insured (38.6%). Encounter acuity was most commonly ESI level 3 (51.8%), and most patients were discharged (65.4%). Patients had an active MyChart account at arrival for 51.5% of encounters. During the study, 17.4% of patients logged into MyChart during their ED stay, 14.1% viewed test results, and 2.5% viewed clinical notes. The Table presents population characteristics stratified by real-time portal activity and missingness rates.

**Table.** Characteristics of Patients Who Logged Into and Viewed Results and Clinical Notes on the Patient Portal

Characteristic	No. of patients (%)						
	Overall	Logged into patient portal		Viewed test results		Viewed clinical notes	
		Yes	No	Yes	No	Yes	No
No. of patients	1 280 924	223 491 (17.4)	1 057 433 (82.6)	180 747 (14.1)	1 100 177 (85.9)	31 640 (2.5)	1 249 284 (97.5)
Age group, y							
18-34	360 005 (28.1)	65 673 (29.4)	294 332 (27.8)	53 323 (29.5)	306 682 (27.9)	8896 (28.1)	351 109 (28.1)
35-49	282 902 (22.1)	53 127 (23.8)	229 775 (21.7)	43 232 (23.9)	239 670 (21.8)	7095 (22.4)	275 807 (22.1)
50-64	292 596 (22.8)	47 211 (21.1)	245 385 (23.2)	37 277 (20.6)	255 319 (23.2)	6281 (19.9)	286 315 (22.9)
65-84	280 959 (21.9)	47 471 (21.2)	233 488 (22.1)	38 016 (21.0)	242 943 (22.1)	7548 (23.9)	273 411 (21.9)
≥85	64 462 (5.0)	10 009 (4.5)	54 453 (5.1)	8899 (4.9)	55 563 (5.1)	1820 (5.8)	62 642 (5.0)
Disposition							
Admitted	364 463 (28.5)	74 936 (33.5)	289 527 (27.4)	62 437 (34.5)	302 026 (27.5)	12 598 (39.8)	351 865 (28.2)
Discharged	837 361 (65.4)	142 715 (63.9)	694 646 (65.7)	115 128 (63.7)	722 233 (65.6)	18 248 (57.7)	819 113 (65.6)
Other^a^	79 100 (6.2)	5840 (2.6)	73 260 (6.9)	3182 (1.8)	75 918 (6.9)	794 (2.5)	78 306 (6.3)
Emergency Severity Index							
1 (highest)	12 502 (1.0)	819 (0.4)	11 683 (1.2)	616 (0.4)	11 886 (1.2)	127 (0.4)	12 375 (1.1)
2	286 007 (22.3)	52 885 (25.2)	233 122 (23.5)	43 830 (25.9)	242 177 (23.5)	8028 (26.7)	277 979 (23.7)
3	664 014 (51.8)	132 213 (63.0)	531 801 (53.7)	109 096 (64.6)	554 918 (53.8)	18 979 (63.1)	645 035 (55.1)
4	220 861 (17.2)	22 966 (10.9)	197 895 (20.0)	15 137 (9.0)	205 724 (19.9)	2853 (9.5)	218 008 (18.6)
5 (lowest)	17 513 (1.4)	853 (0.4)	16 660 (1.7)	275 (0.2)	17 238 (1.7)	103 (0.3)	17 410 (1.5)
Missing	80 027 (6.2)	13 755 (6.6)	66 272 (6.7)	11 793 (7.0)	68 234 (6.6)	1550 (5.2)	78 477 (6.7)
Race and ethnicity							
American Indian or Alaska Native	8311 (0.6)	1262 (0.6)	7049 (0.7)	955 (0.5)	7356 (0.7)	195 (0.6)	8116 (0.7)
Asian	46 812 (3.7)	13 523 (6.1)	33 289 (3.2)	10 417 (5.8)	36 395 (3.3)	2014 (6.4)	44 798 (3.6)
Black	229 993 (18.0)	25 200 (11.3)	204 793 (19.4)	18 602 (10.3)	211 391 (19.3)	2971 (9.4)	227 022 (18.2)
Native Hawaiian or Pacific Islander	5620 (0.4)	1050 (0.5)	4570 (0.4)	716 (0.4)	4904 (0.4)	147 (0.5)	5473 (0.4)
Hispanic	136 741 (10.7)	23 464 (10.8)	113 277 (11.1)	16 775 (9.5)	119 966 (11.3)	2438 (8.1)	134 303 (11.2)
White	852 025 (66.5)	159 258 (71.4)	692 767 (65.8)	133 610 (74.0)	718 415 (65.6)	23 755 (75.3)	828 270 (66.6)
Other race	127 534 (10.0)	23 659 (10.6)	103 875 (9.8)	17 030 (9.4)	110 504 (10.0)	2717 (8.6)	124 817 (10.0)
Missing	51 140 (4.0)	5686 (2.6)	45 454 (4.5)	3927 (2.2)	47 213 (4.5)	1471 (4.9)	49 669 (4.1)
Insurance type							
Commercial	494 809 (38.6)	105 912 (47.5)	388 897 (36.9)	85 439 (47.4)	409 370 (37.2)	13 830 (43.9)	480 979 (38.6)
Medicaid	330 320 (25.8)	47 063 (21.1)	283 257 (26.9)	38 854 (21.6)	291 466 (26.6)	6723 (21.3)	323 597 (26.0)
Medicare	323 970 (25.3)	56 132 (25.2)	267 838 (25.4)	46 060 (25.6)	277 910 (25.3)	8950 (28.4)	315 020 (25.3)
Self-pay	62 300 (4.9)	3685 (1.7)	58 615 (5.6)	2585 (1.4)	59 715 (5.4)	537 (1.7)	61 763 (5.0)
Other^b^	66 181 (5.2)	9995 (4.5)	56 186 (5.3)	7125 (4.0)	59 056 (5.4)	1485 (4.7)	64 696 (5.2)
Missing	3344 (0.3)	704 (0.3)	2640 (0.3)	684 (0.4)	2660 (0.2)	115 (0.4)	3229 (0.3)
Language							
English	1 168 579 (91.2)	209 015 (93.6)	959 564 (91.2)	169 861 (94.0)	998 718 (91.2)	29 673 (93.8)	1 138 906 (91.5)
Spanish	69 786 (5.4)	7604 (3.4)	62 182 (5.9)	5371 (3.0)	64 415 (5.9)	877 (2.8)	68 909 (5.5)
Other	37 520 (2.9)	6715 (3.0)	30 805 (2.9)	5388 (3.0)	32 132 (2.9)	1076 (3.4)	36 444 (2.9)
Missing	5039 (0.4)	157 (0.1)	4882 (0.5)	127 (0.1)	4912 (0.4)	14 (0.0)	5025 (0.4)
Patient portal activation status							
Activated at arrival	659 700 (51.5)	209 775 (93.9)	449 925 (42.5)	171 099 (94.7)	488 601 (44.4)	29 816 (94.2)	629 884 (50.4)
Sex							
Female	685 392 (53.5)	141 703 (63.4)	543 689 (51.4)	116 845 (64.7)	568 547 (51.7)	19 317 (61.1)	666 075 (53.3)
Male	595 072 (46.5)	81 761 (36.6)	513 311 (48.6)	63 880 (35.3)	531 192 (48.3)	12 321 (38.9)	582 751 (46.7)
Missing	460 (0.0)	27 (0.0)	433 (0.0)	22 (0.0)	438 (0.0)	2 (0.0)	458 (0.0)

^a^ Includes patients who left without being seen, before the completion of care, or against medical advice or who immediately went to labor and delivery.

^b^ Includes workers’ compensation and nonclassified payers.

**Figure 1.**
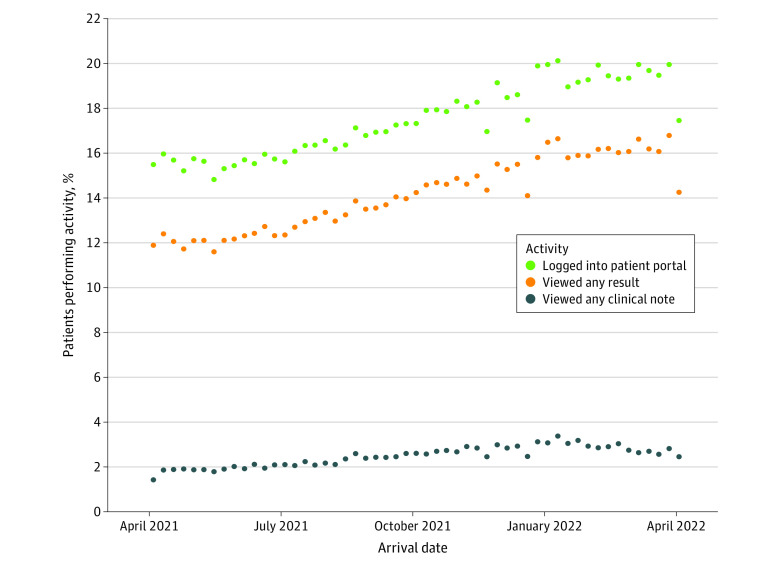
Temporal Trends of Real-Time Emergency Department Patient Portal Use

Figure 1 depicts the combined temporal trends of each portal use activity across all sites, showing increasing rates of all 3 activities, though a larger increase was observed in logging in and viewing results than viewing clinical notes. Trend plots stratified by site are available in eFigure 1 in Supplement 1. Similar usage increases were observed in the pooled models’ effects evaluating the associations between time and each MyChart activity. The weekly ORs for logging in, viewing results, and viewing notes were 1.00607, 1.00961, and 1.00919, respectively; extrapolating from weekly ORs to 51 weeks, assuming a linear effect throughout the study period, patients had a higher odds of logging into MyChart (OR, 1.36; 95% CI, 1.19-1.56), viewing results (OR, 1.63; 95% CI, 1.30-2.04), and viewing clinical notes (OR, 1.60; 95% CI, 1.19-2.15) at the end of the study compared with the beginning.

**Figure 2.**
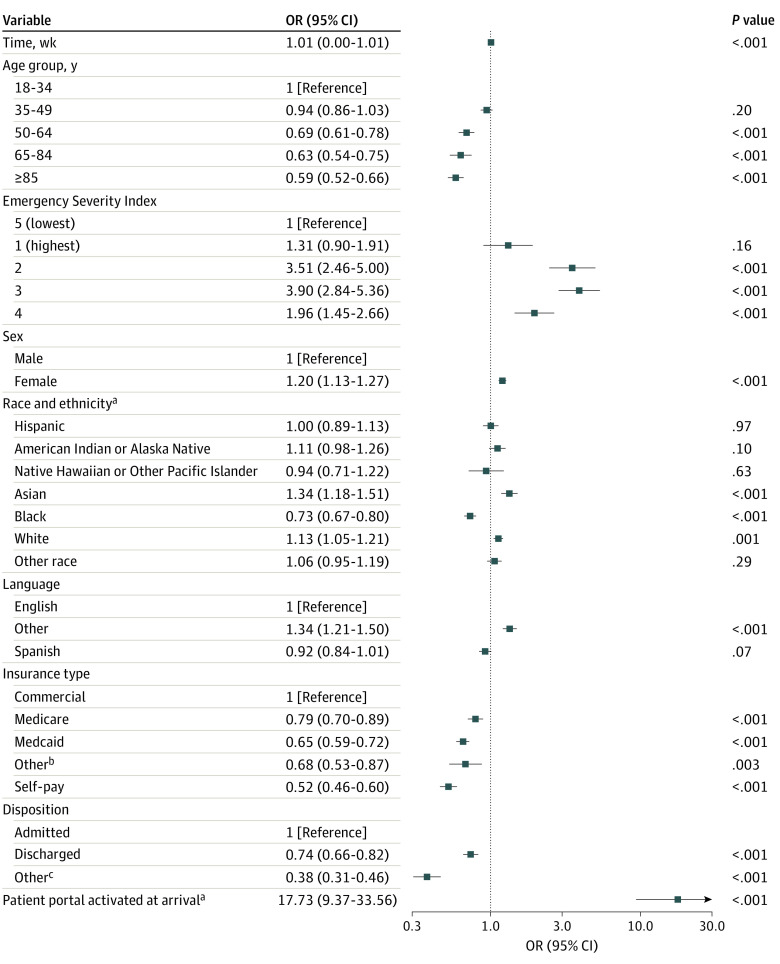
Pooled Odds of Logging Into the Patient Portal During an Emergency Department Encounter ^a^Represents dichotomous variables that are referenced against the inverse of themselves (ie, not Hispanic, no activated portal account). ^b^Includes workers’ compensation and nonclassified payers. ^c^Includes patients who left without being seen, before the completion of care, or against medical advice or who immediately went to labor and delivery.

**Figure 3.**
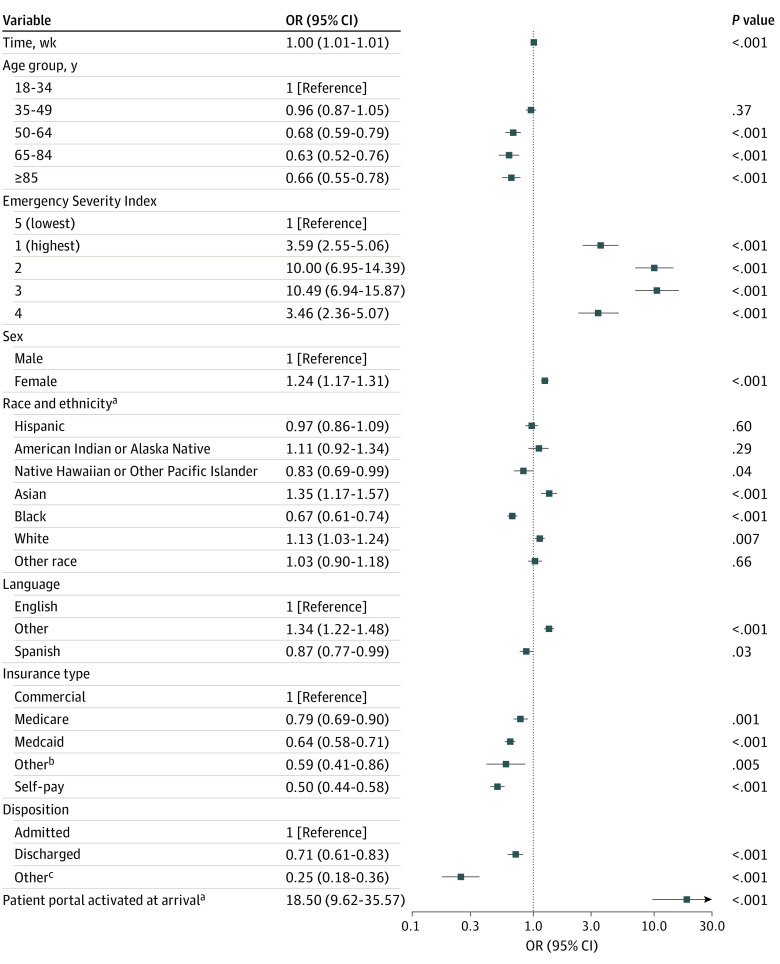
Pooled Odds of Viewing Test Results on the Patient Portal During an Emergency Department Encounter ^a^Represents dichotomous variable that is referenced against the inverse of itself (ie, not Hispanic, no activated portal account). ^b^Includes workers’ compensation and nonclassified payers. ^c^Includes patients who left without being seen, before the completion of care, or against medical advice or who immediately went to labor and delivery.

**Figure 4.**
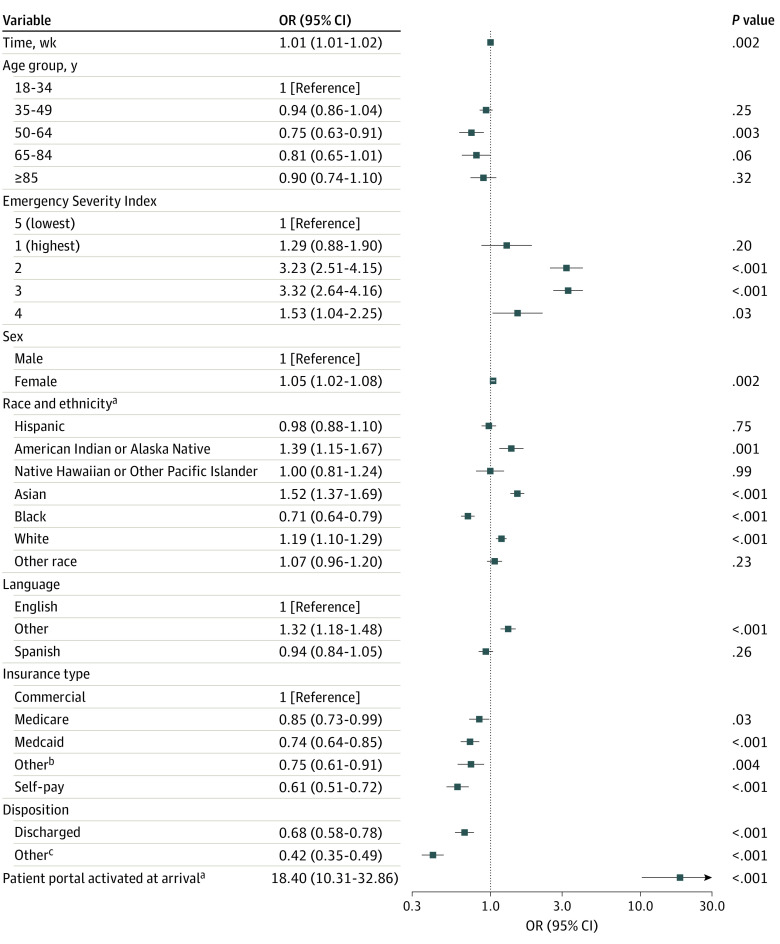
Pooled Odds of Viewing Clinical Notes on the Patient Portal During an Emergency Department Encounter ^a^Represents dichotomous variable that is referenced against the inverse of itself (ie, not Hispanic, no activated portal account). ^b^Includes workers’ compensation and nonclassified payers. ^c^Includes patients who left without being seen, before the completion of care, or against medical advice or who immediately went to labor and delivery.

Pooled models also revealed patient and clinical characteristics associated with real-time portal use (Figure 2, Figure 3, and Figure 4). Patients who arrived at the ED with an active portal account had higher odds of logging in (OR, 17.73; 95% CI, 9.37-33.56), viewing results (OR, 18.50; 95% CI, 9.62-35.57), and viewing clinical notes (OR, 18.40; 95% CI, 10.31-32.86) than patients who arrived without an account. Across all models, patients designated with higher acuity scores (ESI 2 or 3) had more than 3 times higher odds of logging in or reviewing notes and more than 10 times higher odds of viewing results than low-acuity patients (ESI 5). All 3 models indicated that patients who were male, Black, or without commercial health insurance had lower odds of logging in, viewing results, and viewing clinical notes. Patients who were older had lower odds of logging in and viewing results. Patients who were White or Asian or who preferred neither English nor Spanish (ie, other language category) had higher odds across all usage categories. Patients with a Spanish language preference had lower odds of viewing results. Older patients had lower odds of logging in or viewing results but nonsignificant results for viewing clinical notes. Tabular pooled ORs, 95% CIs, and prediction intervals are presented in eTables 2 to 4 in Supplement 1. Single-site ORs and 95% CIs are plotted in eFigures 2 to 4 in Supplement 1.

Prediction intervals were consistent in direction and scale with 95% CIs for most variables with a clinically and statistically significant association with all 3 MyChart activities, including time, ESI, legal sex, Black race, Medicaid or self-pay insurance status, other preferred language, disposition, and MyChart activation status at arrival. There were several variables where prediction intervals suggested that repeated trials might find contradictory ORs, including Spanish language preference, age, Hispanic ethnicity, Asian race, Native Hawaiian or other Pacific Islander race, Medicare status, and age.

As shown in eFigure 1 in Supplement 1, Stanford Health Care had a substantially higher MyChart activation rate than the other sites. We repeated our analyses excluding Stanford’s data as a sensitivity analysis to ensure that this intersite difference did not change the results. The resulting models had similar effect sizes and significance patterns.

## Discussion

Our cross-sectional study is, to our knowledge, the largest to date of real-time patient portal use among ED patients. Using data from 36 geographically distributed academic and community EDs across the US, we conducted a robust semifederated statistical analysis to measure trends and disparities in logging into the portal, viewing test results, and viewing clinical notes during ED encounters. We observed a consistent increase in the proportion of patients who performed all 3 activities during the study period. Despite this growth, there were differences in portal access and use across multiple demographic and clinical variables.

We observed substantial growth in the percentage of patients who accessed the portal during ED encounters. Prior work has highlighted similar growth in general portal use^1^ due in part to growing reliance on digital health care delivery and regulatory factors.^28^ We similarly observed an increasing proportion of patients who viewed their test results in real time. We hypothesize that the Cures Act, which requires that nearly all test results be immediately available to patients upon request, may have contributed to this trend. Research in the outpatient setting has found that many patients now access sensitive test results before their clinicians do.^4,29^ Improved information availability promotes opportunities to increase engagement and supports shared decision-making among patients, caregivers, and clinicians.^29,30,31,32^ Further research is necessary to better understand the effects of immediate test result availability in acute care settings, including the ED. Prior work found that a subset of patients who reviewed abnormal test results experienced increased worry or emotional distress.^8,9^ Most patients in our study who accessed test results received care for conditions classified as ESI 2 or 3, which are often associated with abnormal results. Ideal communication strategies, such as precounseling before ordering a test, may differ in an ED compared with an ambulatory setting. Further research is needed to establish best practices to support patients in reviewing and using electronic health information.

Despite growth in portal use, our pooled models identified several demographic and clinical disparities. We noted lower odds of portal access and use among patients who are male, Black, or without commercial insurance. These results are consistent with prior analyses of general portal use.^33^ There were heterogeneous findings regarding the association between Hispanic ethnicity and Spanish language preference with portal use across sites, which may be related to varied penetration of Spanish-language features in different MyChart configurations or varying levels of infrastructure to support Spanish speakers at different institutions. Further studies are needed to better understand these findings.

Observed portal use differences may reflect cultural preferences, but the literature suggests that portal access may represent a bottleneck to use. Richwine and colleagues found that Black and Hispanic patients were offered access to portals at a significantly lower rate than White patients, but usage rates were similar among enrolled patients from both groups.^1,34^ Our analysis found that patients arriving at the ED with an active portal account had 17.73 to 18.50 times greater odds of accessing the patient portal and viewing results, respectively, during the encounter, suggesting that the portal was mostly used by patients with existing access to care. Future research should compare detailed usage patterns between patients with activated accounts at arrival vs those who activated an account during their ED stay.

Emergency departments serve as safety-net clinics for patients from medically underserved social and demographic groups without access to care. Facilitating portal enrollment during ED encounters represents an underused opportunity to address enrollment inequities. For example, care navigators or registration staff could educate patients on portal use, support real-time enrollment, and provide technical support during waiting and boarding times. Several initiatives have improved portal enrollment among medically underserved populations by providing hands-on support in the ED to enroll and educate patients about portal functionality.^35,36^ Interestingly, our results indicate substantial variability in MyChart activation rates among sites, especially at Stanford Health Care, which we theorize is associated with both a digitally literate population and the institution’s long history of supporting patient portal efforts. Despite Stanford as an outlier, most sites had similar features associated with higher odds of portal use. Our results suggest that there may be ED-based enrollment gains to be made, even within institutions with a high baseline activation rate.

### Limitations

This study has some limitations. All participating sites used Epic’s MyChart patient portal, and unexplored vendor differences may contribute to variation in portal enrollment or use at other health systems. Some patients may have reviewed relevant ED results or results from other visits via linked accounts in MyChart across health systems, which we were unable to account for. Our study relied on a semifederated approach that combined results across institutions. This approach allowed us to enroll multiple sites while maintaining consistency across data elements, but we were unable to obtain patient-level data to evaluate reasons for patient portal use or nonuse. Future studies should investigate patient attitudes and perceptions toward real-time patient portal use in the ED.

There is wide variance in MyChart enrollment across institutions with regard to policy, culture, and individual employee practice. Future qualitative or survey-based studies could describe interinstitutional variability in portal enrollment practices. Such studies might also explain our heterogenous results for certain variables. There may be a population of patients who, due to varying chronic complexity of care, acuity, and patient preferences, may benefit more than others from accessing the portal in real time, though we did not account for this complexity beyond inclusion of the ESI score. Future studies could normalize portal activity to the number and complexity of test results or notes that are available for review.

## Conclusions

This cross-sectional study measured trends in real-time patient portal activity during ED encounters and demographic differences between portal users and nonusers across 36 geographically distributed community and academic EDs. We observed a temporal increase in patients logging into the portal, viewing test results, and viewing clinical notes. Despite this increase, patients who were male, Black, or without commercial insurance had a lower odds of using the portal during their encounter. Because EDs serve as the primary care environment for many patients, they have a unique opportunity to support interested patients in portal enrollment, access, and use.

## Notes

Supplement 1. **eTable 1.** Site Characteristics **eTable 2.** Pooled Odds Ratios and Prediction Intervals for Odds of Logging Into MyChart in Real Time During an ED Encounter **eTable 3.** Pooled Odds Ratios and Prediction Intervals for Odds of Reviewing Results in MyChart in Real Time During an ED Encounter **eTable 4.** Pooled Odds Ratios and Prediction Intervals for Odds of Reviewing Notes in MyChart in Real Time During an ED Encounter **eFigure 1.** Temporal Trends of Portal Activity Use (Stratified by Site) **eFigure 2.** Results of Multivariable Model Showing Odds of Logging Into MyChart as a Function of Patient Factors (Stratified by Site) **eFigure 3.** Results of Multivariable Model Showing Odds of Viewing Results in MyChart as a Function of Patient Factors (Stratified by Site) **eFigure 4.** Results of Multivariable Model Showing Odds of Viewing Clinical Notes in MyChart as a Function of Patient Factors (Stratified by Site)

Supplement 2.
Data Sharing Statement
