# Supplementary material for: Real-Time Electronic Patient Portal Use Among Emergency Department Patients
Source: JAMA Netw Open. 2024 May 3;7(5):e249831. doi: 10.1001/jamanetworkopen.2024.9831 (PMC11069088; doi:10.1001/jamanetworkopen.2024.9831)
Supplement: Supplement 1. — eTable 1. Site Characteristics eTable 2. Pooled Odds Ratios and Prediction Intervals for Odds of Logging Into MyChart in Real Time During an ED Encounter eTable 3. Pooled Odds Ratios and Prediction Intervals for Odds of Reviewing Results in MyChart in Real Time During an ED Encounter eTable 4. Pooled Odds Ratios and Prediction Intervals for Odds of Reviewing Notes in MyChart in Real Time During an ED Encounter eFigure 1. Temporal Trends of Portal Activity Use (Stratified by Site) eFigure 2. Results of Multivariable Model Showing Odds of Logging Into MyChart as a Function of Patient Factors (Stratified by Site) eFigure 3. Results of Multivariable Model Showing Odds of Viewing Results in MyChart as a Function of Patient Factors (Stratified by Site) eFigure 4. Results of Multivariable Model Showing Odds of Viewing Clinical Notes in MyChart as a Function of Patient Factors (Stratified by Site) [file jamanetwopen-e249831-s001.pdf]

Supplemental Online Content

Turer RW, McDonald SA, Lehmann CU, et al. Real-time electronic patient portal use among emergency department patients. *JAMA Netw Open*. 2024;7(5):e249831. doi:10.1001/jamanetworkopen.2024.9831

eTable 1. Site Characteristics

eTable 2. Pooled Odds Ratios and Prediction Intervals for Odds of Logging Into MyChart in Real Time During an ED Encounter

eTable 3. Pooled Odds Ratios and Prediction Intervals for Odds of Reviewing Results in MyChart in Real Time During an ED Encounter

eTable 4. Pooled Odds Ratios and Prediction Intervals for Odds of Reviewing Notes in MyChart in Real Time During an ED Encounter

eFigure 1. Temporal Trends of Portal Activity Use (Stratified by Site)

eFigure 2. Results of Multivariable Model Showing Odds of Logging Into MyChart as a Function of Patient Factors (Stratified by Site)

eFigure 3. Results of Multivariable Model Showing Odds of Viewing Results in MyChart as a Function of Patient Factors (Stratified by Site)

eFigure 4. Results of Multivariable Model Showing Odds of Viewing Clinical Notes in MyChart as a Function of Patient Factors (Stratified by Site)

This supplemental material has been provided by the authors to give readers additional information about their work.

© 2024 Turer RW et al. *JAMA Network Open.*

eTable 1. Site Characteristics

Site	State	# Academic EDs	# Community EDs	Total Volume
Kettering Health	OH	2	13	319803
Mass General Brigham	MA	2	7	395974
Stanford Health Care	CA	1	0	67270
University of California San Diego	CA	2	0	84694
University Of Washington	WA	2	1	114450
UT Southwestern Medical Center	TX	1	0	53109
Vanderbilt University Medical Center	TN	1	1	97640
Yale New Haven Health	CT	1	2	171070
**Total**		12	24	1304010

© 2024 Turer RW et al. *JAMA Network Open.*

eTable 2. Pooled Odds Ratios and Prediction Intervals for Odds of Logging Into MyChart in Real Time During an ED Encounter

Parameter	OR	95% CI	P Value	Prediction Interval
Intercept	0.011	0.00 - 0.02	<0.001	0.00 - 0.11
Time (Study Week)	1.006	1.00 - 1.01	<0.001	1.00 - 1.01
Age(35 - 49)	0.942	0.86 - 1.03	0.196	0.73 - 1.22
Age(50 - 64)	0.691	0.61 - 0.78	<0.001	0.49 - 0.98
Age(65 - 84)	0.632	0.54 - 0.75	<0.001	0.39 - 1.03
Age(85 or older)	0.586	0.52 - 0.66	<0.001	0.42 - 0.82
ESI(1)	1.311	0.90 - 1.91	0.159	0.46 - 3.70
ESI(2)	3.507	2.46 - 5.00	<0.001	1.28 - 9.57
ESI(3)	3.903	2.84 - 5.36	<0.001	1.61 - 9.48
ESI(4)	1.961	1.45 - 2.66	<0.001	0.84 - 4.56
Sex(Female)	1.201	1.13 - 1.27	<0.001	1.02 - 1.41
Hispanic	1.003	0.89 - 1.13	0.967	0.72 - 1.39
American Indian or Alaska Native	1.112	0.98 - 1.26	0.097	0.90 - 1.38
Native Hawaiian or Pacific Islander	0.935	0.71 - 1.22	0.627	0.49 - 1.78
Asian	1.336	1.18 - 1.51	<0.001	0.96 - 1.86
Black	0.731	0.67 - 0.80	<0.001	0.58 - 0.92
White	1.126	1.05 - 1.21	0.001	0.95 - 1.34
Other Race	1.061	0.95 - 1.19	0.292	0.80 - 1.40
Language(Other)	1.343	1.21 - 1.50	<0.001	1.02 - 1.77
Language(Spanish)	0.919	0.84 - 1.01	0.074	0.74 - 1.15
Insurance(Medicare)	0.792	0.70 - 0.89	<0.001	0.56 - 1.11
Insurance(Medicaid)	0.652	0.59 - 0.72	<0.001	0.49 - 0.86
Insurance(Other)	0.680	0.53 - 0.87	0.003	0.33 - 1.41
Insurance(Self-Pay)	0.525	0.46 - 0.60	<0.001	0.37 - 0.75
Disposition(Discharged)	0.736	0.66 - 0.82	<0.001	0.52 - 1.03
Disposition(Other)	0.376	0.31 - 0.46	<0.001	0.20 - 0.70
MyChart Activated at Arrival	17.731	9.37 - 33.56	<0.001	2.62 - 120.09

© 2024 Turer RW et al. *JAMA Network Open.*

eTable 3. Pooled Odds Ratios and Prediction Intervals for Odds of Reviewing Results in MyChart in Real Time During an ED Encounter

Parameter	OR	95% CI	P Value	Prediction Interval
Intercept	0.002	0.00 - 0.00	<0.001	0.00 - 0.01
Time (Study Week)	1.010	1.01 - 1.01	<0.001	1.00 - 1.02
Age(35 - 49)	0.956	0.87 - 1.05	0.368	0.72 - 1.27
Age(50 - 64)	0.681	0.59 - 0.79	<0.001	0.44 - 1.05
Age(65 - 84)	0.629	0.52 - 0.76	<0.001	0.36 - 1.11
Age(85 or older)	0.656	0.55 - 0.78	<0.001	0.40 - 1.08
ESI(1)	3.593	2.55 - 5.06	<0.001	1.64 - 7.89
ESI(2)	9.998	6.95 - 14.39	<0.001	4.00 - 24.99
ESI(3)	10.494	6.94 - 15.87	<0.001	3.59 - 30.68
ESI(4)	3.458	2.36 - 5.07	<0.001	1.31 - 9.13
Sex(Female)	1.239	1.17 - 1.31	<0.001	1.05 - 1.46
Hispanic	0.968	0.86 - 1.09	0.596	0.69 - 1.36
American Indian or Alaska Native	1.109	0.92 - 1.34	0.285	0.71 - 1.73
Native Hawaiian or Pacific Islander	0.827	0.69 - 0.99	0.041	0.59 - 1.16
Asian	1.354	1.17 - 1.57	<0.001	0.90 - 2.03
Black	0.669	0.61 - 0.74	<0.001	0.53 - 0.85
White	1.132	1.03 - 1.24	0.007	0.90 - 1.42
Other Race	1.030	0.90 - 1.18	0.664	0.74 - 1.44
Language(Other)	1.345	1.22 - 1.48	<0.001	1.08 - 1.68
Language(Spanish)	0.874	0.77 - 0.99	0.031	0.65 - 1.18
Insurance(Medicare)	0.787	0.69 - 0.90	0.001	0.53 - 1.17
Insurance(Medicaid)	0.641	0.58 - 0.71	<0.001	0.49 - 0.84
Insurance(Other)	0.592	0.41 - 0.86	0.005	0.20 - 1.73
Insurance(Self-Pay)	0.503	0.44 - 0.58	<0.001	0.35 - 0.72
Disposition(Discharged)	0.712	0.61 - 0.83	<0.001	0.46 - 1.11
Disposition(Other)	0.251	0.18 - 0.36	<0.001	0.09 - 0.73
MyChart Activated at Arrival	18.499	9.62 - 35.57	<0.001	2.61 - 131.23

© 2024 Turer RW et al. *JAMA Network Open.*

eTable 4. Pooled Odds Ratios and Prediction Intervals for Odds of Reviewing Notes in MyChart in Real Time During an ED Encounter

Parameter	OR	95% CI	P Value	Prediction Interval
Intercept	0.001	0.00 - 0.00	<0.001	0.00 - 0.02
Time (Study Week)	1.009	1.00 - 1.02	0.002	0.99 - 1.03
Age(35 - 49)	0.945	0.86 - 1.04	0.253	0.73 - 1.22
Age(50 - 64)	0.753	0.63 - 0.91	0.003	0.44 - 1.28
Age(65 - 84)	0.811	0.65 - 1.01	0.064	0.43 - 1.53
Age(85 or older)	0.904	0.74 - 1.10	0.319	0.53 - 1.54
ESI(1)	1.291	0.88 - 1.90	0.196	0.63 - 2.63
ESI(2)	3.228	2.51 - 4.15	<0.001	2.10 - 4.95
ESI(3)	3.318	2.64 - 4.16	<0.001	2.37 - 4.65
ESI(4)	1.531	1.04 - 2.25	0.030	0.63 - 3.69
Sex(Female)	1.048	1.02 - 1.08	0.002	1.02 - 1.08
Hispanic	0.982	0.88 - 1.10	0.750	0.76 - 1.27
American Indian or Alaska Native	1.387	1.15 - 1.67	0.001	1.15 - 1.67
Native Hawaiian or Pacific Islander	1.001	0.81 - 1.24	0.989	0.81 - 1.24
Asian	1.520	1.37 - 1.69	<0.001	1.37 - 1.69
Black	0.711	0.64 - 0.79	<0.001	0.60 - 0.84
White	1.189	1.10 - 1.29	<0.001	1.10 - 1.29
Other Race	1.073	0.96 - 1.20	0.228	0.92 - 1.25
Language(Other)	1.323	1.18 - 1.48	<0.001	1.06 - 1.65
Language(Spanish)	0.939	0.84 - 1.05	0.255	0.79 - 1.11
Insurance(Medicare)	0.847	0.73 - 0.99	0.032	0.55 - 1.30
Insurance(Medicaid)	0.736	0.64 - 0.85	<0.001	0.50 - 1.08
Insurance(Other)	0.746	0.61 - 0.91	0.004	0.45 - 1.23
Insurance(Self-Pay)	0.609	0.51 - 0.72	<0.001	0.42 - 0.88
Disposition(Discharged)	0.676	0.58 - 0.78	<0.001	0.44 - 1.04
Disposition(Other)	0.415	0.35 - 0.49	<0.001	0.27 - 0.64
MyChart Activated at Arrival	18.402	10.31 - 32.86	<0.001	3.29 - 102.82

© 2024 Turer RW et al. *JAMA Network Open.*

eFigure 1. Temporal Trends of Portal Activity Use (Stratified by Site)

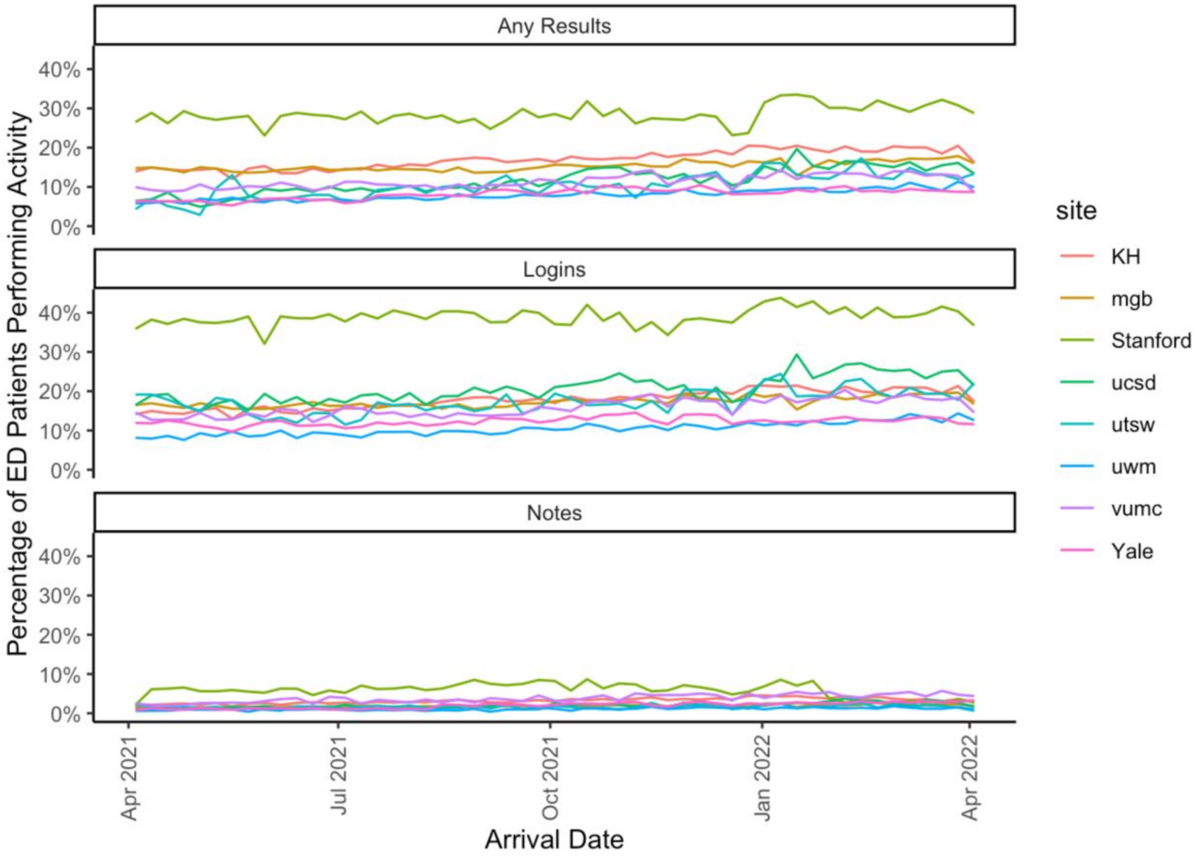

© 2024 Turer RW et al. *JAMA Network Open.*

eFigure 2. Results of Multivariable Model Showing Odds of Logging Into MyChart as a Function of Patient Factors (Stratified by Site).

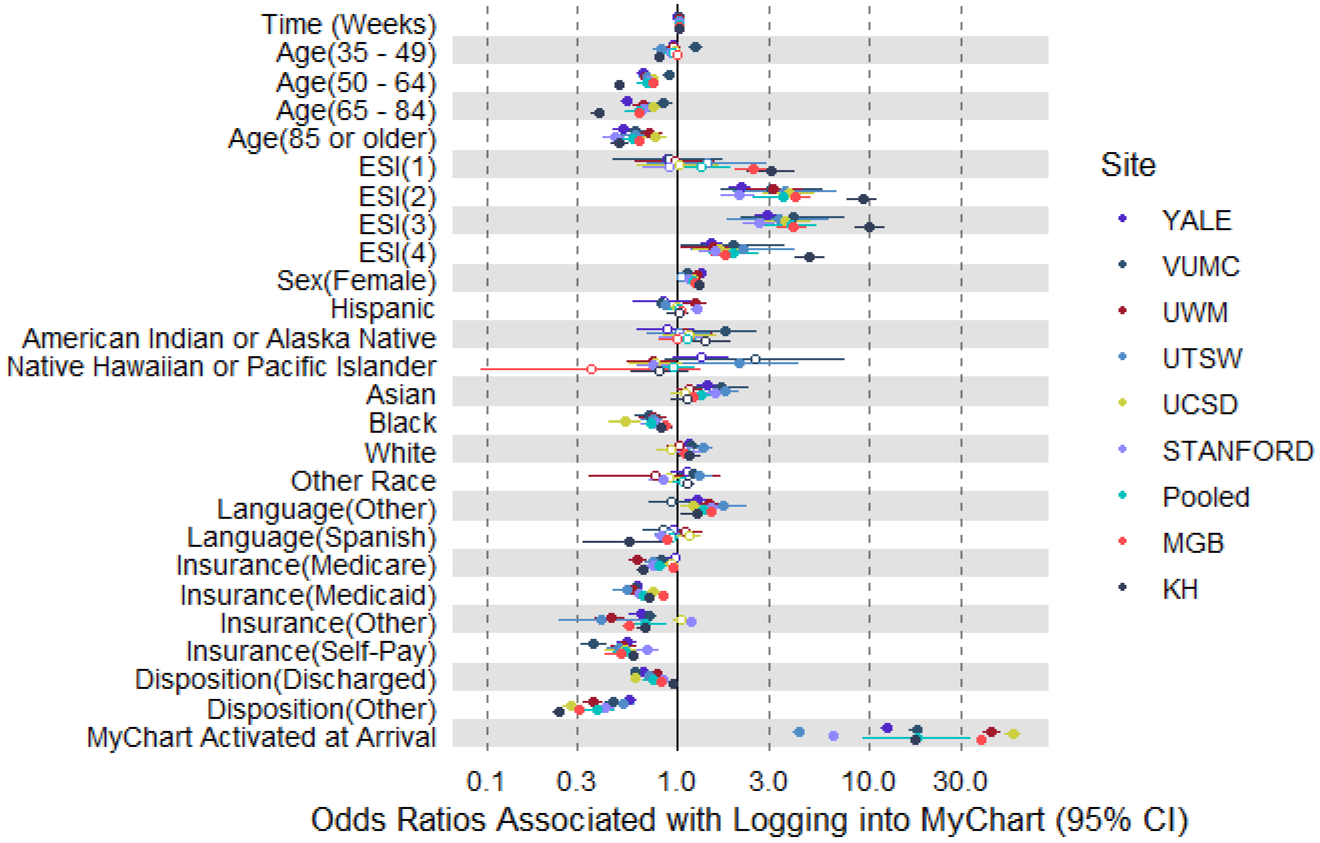

© 2024 Turer RW et al. *JAMA Network Open.*

eFigure 3. Results of Multivariable Model Showing Odds of Viewing Results in MyChart as a Function of Patient Factors (Stratified by Site)

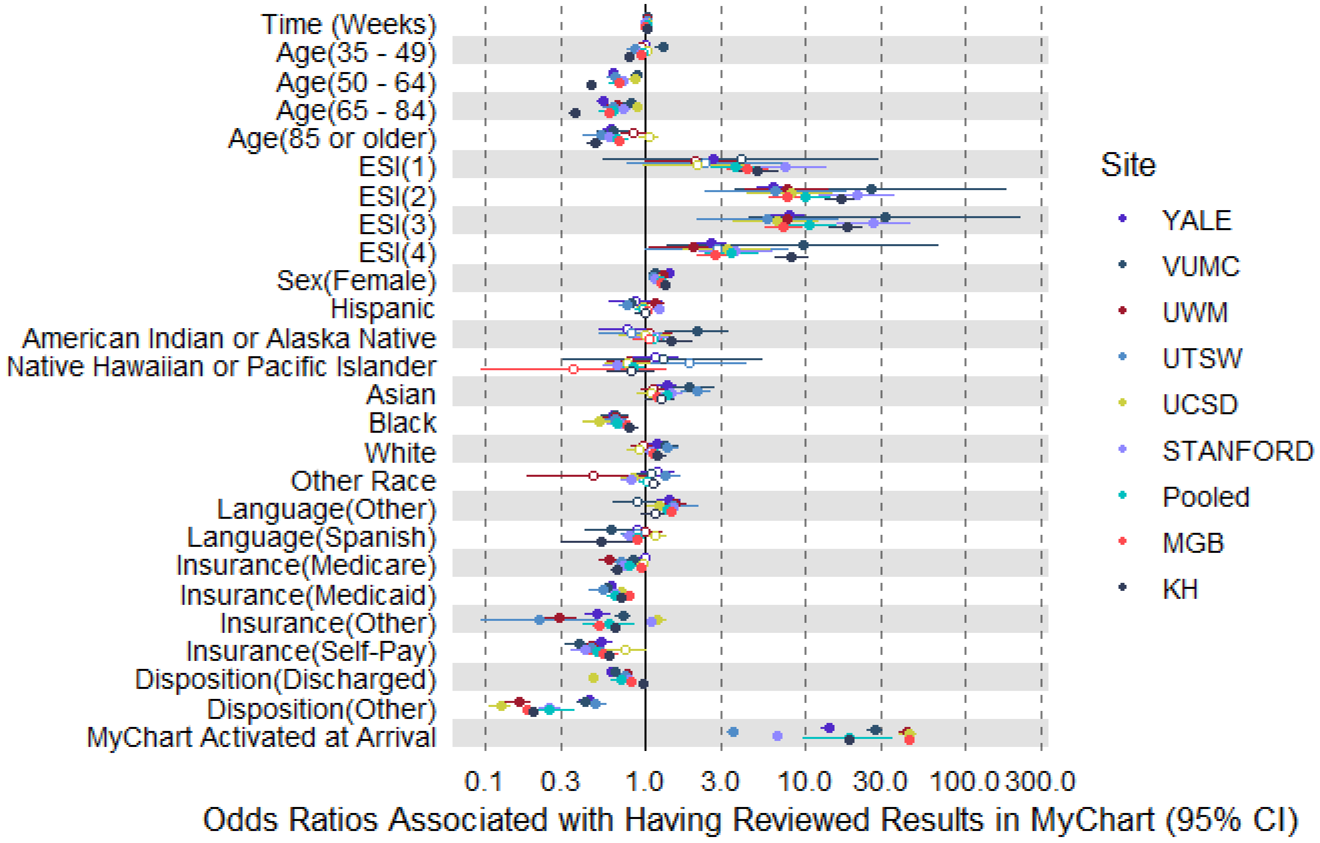

© 2024 Turer RW et al. *JAMA Network Open.*

eFigure 4. Results of Multivariable Model Showing Odds of Viewing Clinical Notes in MyChart as a Function of Patient Factors (Stratified by Site)

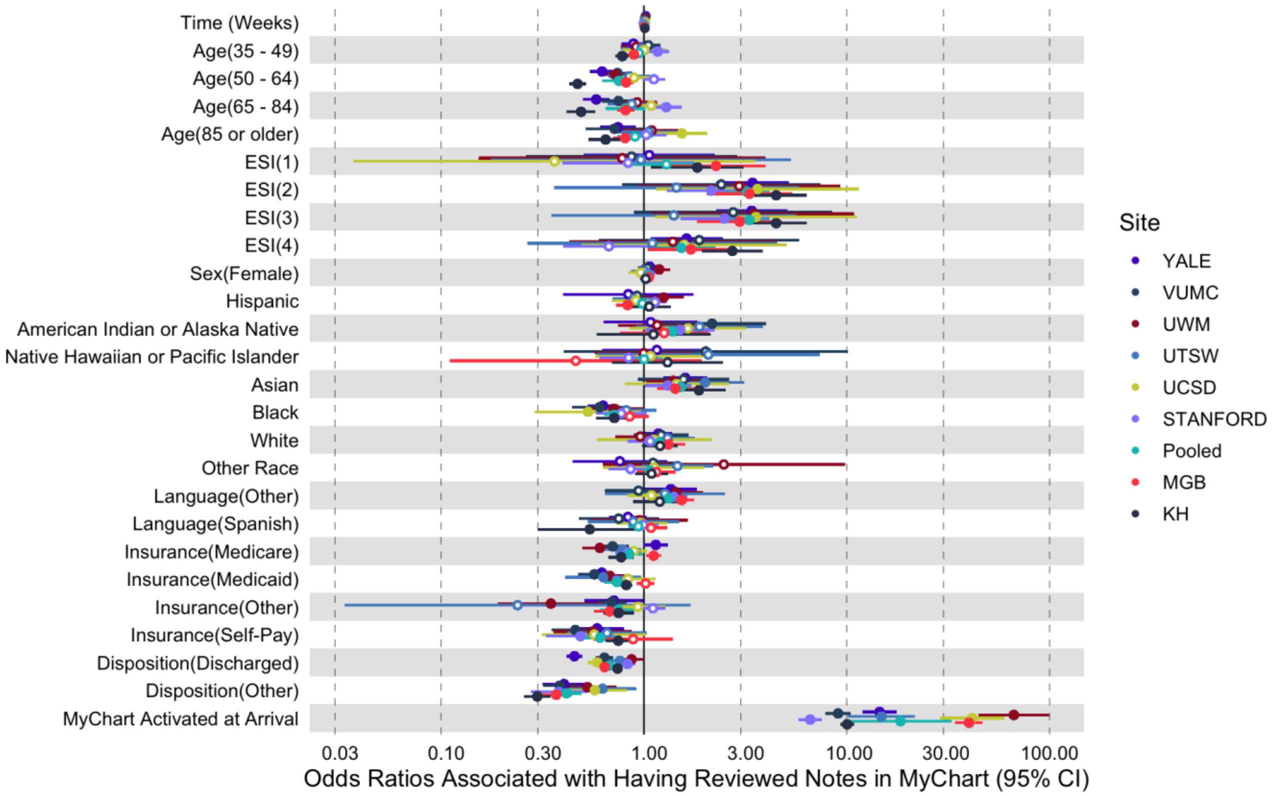

© 2024 Turer RW et al. *JAMA Network Open.*
