# Supplementary material for: Real-Time Electronic Patient Portal Use Among Emergency Department Patients
Source: JAMA Netw Open. 2024 May 3;7(5):e249831. doi: 10.1001/jamanetworkopen.2024.9831 (PMC11069088; doi:10.1001/jamanetworkopen.2024.9831)
Supplement: Supplement 2. — Data Sharing Statement [file jamanetwopen-e249831-s002.pdf]

# Data Sharing Statement

Turer. Real-Time Electronic Patient Portal Use Among Emergency Department Patients. *JAMA Netw Open*. Published May 03, 2024. doi:10.1001/jamanetworkopen.2024.9831

**Data**
**Data available:** No

**Additional Information**
**Explanation for why data not available:** The row-level data for this study include protected health information and are not publicly shareable per our institutional review board.
